# Rapid transition to home omalizumab treatment for chronic spontaneous urticaria during the COVID-19 pandemic: A patient perspective

**DOI:** 10.1016/j.waojou.2021.100587

**Published:** 2021-09-21

**Authors:** Catherine King, Fionnuala Cox, Anne Sloan, Patricia McCrea, J.David Edgar, Niall Conlon

**Affiliations:** a Department of Clinical and Laboratory Immunology, St. James's Hospital, James’s Street, Dublin 8, Ireland; b Department of Immunology, School of Medicine, Trinity College Dublin, Dublin, Ireland

**Keywords:** Chronic urticaria, Omalizumab, Self-administration, Patient reported outcome measure, COVID-19

## Abstract

Efforts to reduce non-urgent hospital attendances during the COVID-19 pandemic have been the focus of much attention from healthcare professionals worldwide. In Ireland, due to funding constraints omalizumab is only available for hospital-based administration. Fifty-eight patients with chronic spontaneous urticaria and angioedema (CSU) receiving omalizumab in our centre were rapidly transitioned to home self-administration at the start of the pandemic. We conducted an anonymised patient survey after 3 months of home therapy with the aim of characterizing the patient experience throughout this period. 41 patients participated in our questionnaire (71% response rate). 93% of patients favored self-injection of omalizumab from home, with respondents citing cost savings, time savings, improved flexibility, fewer hospital visits, and less risk of exposure to COVID-19 infection as particular benefits. Concerns regarding home administration including injecting incorrectly, forgetting a dose, or having a reaction were reported very infrequently. Eighty-three percent (83%) of patients wished to continue with home therapy long-term. This survey highlights broadly positive experiences for patients rapidly transitioning to home omalizumab administration. This data will be useful to inform healthcare funders in decisions regarding patient-centred care in CSU. Facilitating home omalizumab therapy in suitable CSU patients should be strongly considered in the post-pandemic setting.

The disruption of healthcare services due to COVID-19 has led to innovations in service delivery that may have implications for patient care beyond the pandemic. Patients with chronic spontaneous urticaria and angioedema (CSU) have been affected, with a recent international study reporting significant decreases in face-to-face clinical encounters, reduced prescribing of immunosuppressive therapies, and associated flares of CSU in the setting of concurrent COVID-19 infection.^1^

CSU patients with symptoms refractory to high dose antihistamines may benefit from the anti-IgE monoclonal antibody omalizumab.^2^ In Ireland, this medication has been solely available as a hospital-based treatment, despite licensing approval for self-administration being granted in 2018.^3^ Given the exceptional circumstances of the COVID-19 pandemic and the need to limit hospital footfall, the transition from clinic to home-based self-administration of omalizumab was facilitated for patients with CSU in our centre. Patients received at least 3 doses of omalizumab in the hospital setting and subsequently received training in self-injection by nursing staff. After 3 months of home administration, patients were invited to participate in an anonymised, voluntary survey with the aim of assessing acceptability of omalizumab in the home setting and to characterize the patient experience in this period of previously unplanned transition, and to inform future decisions of healthcare funders.

Fifty-eight patients who transitioned to home-based omalizumab treatment were invited to participate in a paper-based or online questionnaire (Supplemental Appendix 1). This survey was partially based on a questionnaire of asthma patients receiving omalizumab.^4^ Ethical approval was obtained from the National Research Ethics Committee for COVID-19-related Research (ID number: 20-NREC-COV-062).

Questionnaire responses were received from 41 of 58 patients who were self-administering (71% response rate). Respondent characteristics are detailed in Table 1. In addition to a substantial €80 statutory day service attendance charge, the majority of patients reported personal costs of at least €10 (66%), with 10% of patients having to spend upwards of €50 per visit. Twenty-four percent (24%) of patients (n = 10) reported over 11 days lost from work per year with regards facilitating dayward attendances. The vast majority of patients reported that greater than one hour of their time would be saved by administering omalizumab at home, with 17% reporting time savings of over 5 hours per administration. 93% of patients agreed with the statement: “I am in favor of injecting omalizumab from home”. Seventy-nine percent (79%) of patients (31 out of 39 respondents, 2 left this question unanswered) indicated that they would have preferred that the option of home administration would have been available to them prior to this emergency.

**Table 1:** Baseline characteristics of respondents.

Baseline Characteristics (n = 41)	
Female sex – n (%)	34 (90)
Age – mean, years (range)	43 (20–79)
Duration of omalizumab therapy – n (%)	
- < 1 year	11 (27)
- 1–4 years	25 (61)
- 5–9 years	5 (12)
Omalizumab dosing intervals – n (%)	
- 3-weekly	7 (17)
- 4-weekly	24 (59)
- 5-weekly	5 (12)
- 6-weekly	5 (12)
Omalizumab dose – n (%)	
- 300 mg	34 (83)
- 450 mg	6 (15)
- 600 mg	1 (2)
Baseline satisfaction with omalizumab in management of symptoms – n (%)	
- Extremely Satisfied	27 (66)
- Satisfied	10 (24)
- Somewhat Satisfied	3 (7)
- Dissatisfied	1 (2)
- Extremely Dissatisfied	0 (0)

High satisfaction was reported in relation to the support available from the clinical team. There was over 95% agreement by respondents that team members took sufficient time with them, were aware of their situation, were contactable when needed, and answered questions well. The training in self-administration was viewed favorably, with 93% of patients stating the number of practice sessions was sufficient, and 91% feeling confident in self-injection after training was received.

Perceived benefits associated with home administration, as well as potential difficulties were explored, with responses summarized in Fig. 1. Financial benefits, time savings and quality of life benefits were addressed and generally agreed upon by respondents. Reduced risk of COVID-19 exposure in the home setting, however, was the advantage which drew the highest level of support from respondents overall, with 38 of 39 respondents (97%, 2 patients leaving this question unanswered) citing this as a particularly important factor. 66% of patients indicated concern over attending hospital as the pandemic escalated. In contrast, concerns relating to the home setting were reported less frequently, with 18% of patients reporting fear of injecting incorrectly (n = 7/40), 13% reporting fear of forgetting a dose (n = 5/38) and 15% reporting fear of having an adverse reaction to omalizumab (n = 6/39).

**Fig. 1:**
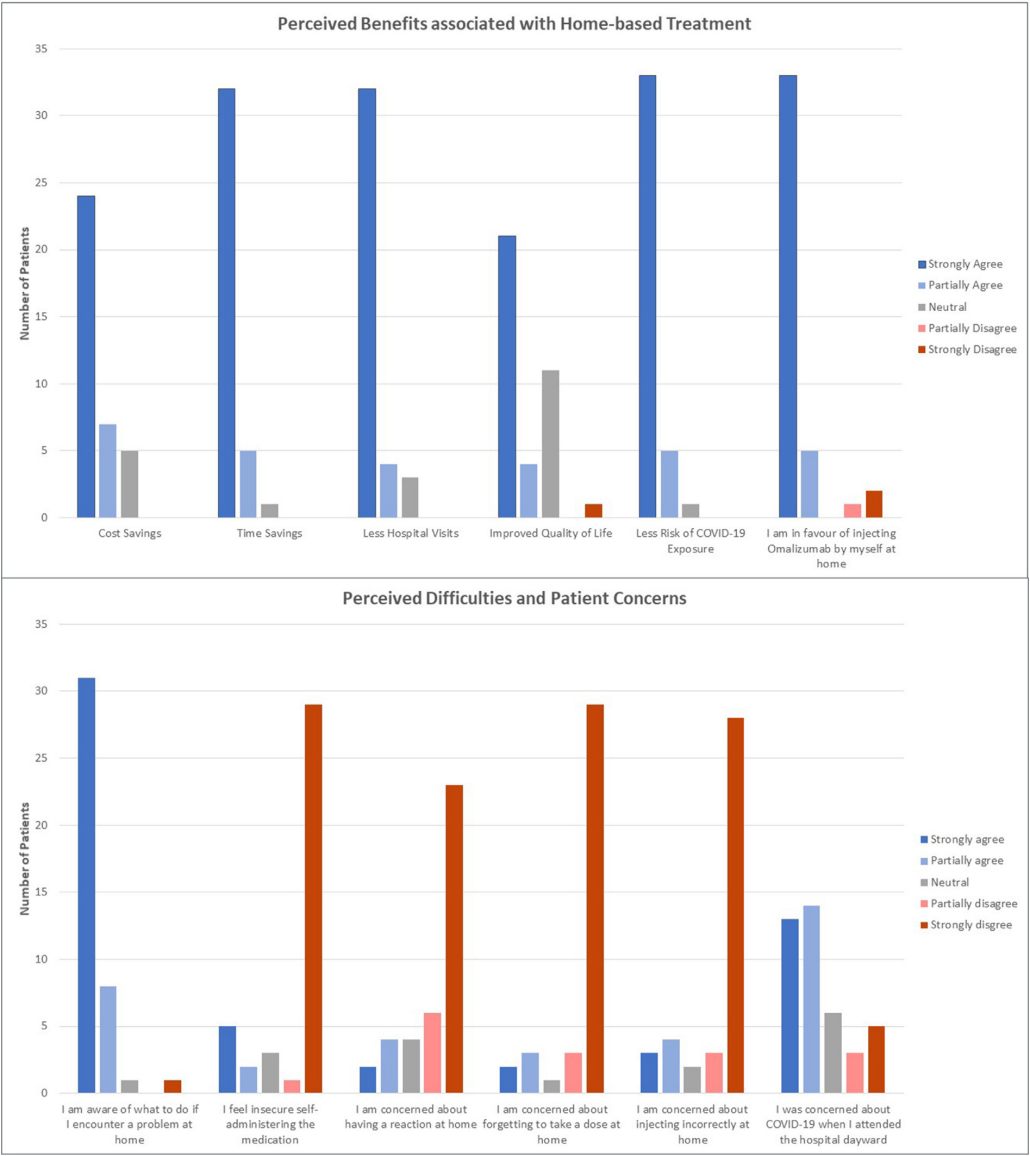
Questionnaire responses addressing perceived benefits to home-based omalizumab administration and perceived difficulties.  Strongly Agree  Partially Agree  Neutral  Partially Disagree  Strongly Disagree.

Eighty-three percent (83%) of patients stated they would prefer to continue home-based treatment in the post-pandemic setting. Amongst the 7 patients who indicated a preference for hospital-based treatment, data suggested this cohort were slightly older (an average age of 48.9 years, versus 41.9 years for the cohort in favor of home-based treatment), were more often on higher doses at baseline (29% on doses above 300 mg, versus 15% in the cohort who preferred home-based treatment), and were less likely to be extremely satisfied with omalizumab at baseline (43%, versus 71% reported by the cohort who preferred home-based treatment), although statistical significance was not observed. This could suggest a less well controlled cohort, more dependent on regular clinical review.

Respondents indicated on a rating scale how likely they would be to recommend home-based omalizumab to others with their condition (0 = definitely would not recommend, 10 = definitely would recommend), with an average rating of 8.88 given. They were also asked to rate their overall experience of injecting omalizumab from home (0 = very bad, 10 = excellent), with an average rating of 9 reported.

Patients could provide an optional final comment at conclusion of the survey. Statements citing time savings as a particular benefit were again seen (n = 5), as were comments stating that respondents felt comfortable self-injecting (n = 6). The benefits of not needing to take time off work (n = 5) or to travel far distances (n = 4) were also highlighted. Four patients stated the current arrangement was beneficial to the clinical team as time could now be allocated to other patients. Eight respondents emphasized a desire for ongoing regular clinical reviews, with 4 patients specifically stating they appreciated the ease with which they could discuss problems with the clinical team during dayward attendances previously.

Overall, this cohort of patients demonstrated high levels of satisfaction with home-based self-administration of omalizumab. A wide range of ages was represented by our respondents, as well as patients who required individualized dosing regimens (Table 1).

Self-administration of omalizumab in patients with CSU has been infrequently described in the medical literature to date. In a study evaluating attitudes of asthmatic patients receiving omalizumab in hospital, only 45% reported a preference for theoretically transitioning to self-injection with a similar proportion also highlighting concerns about the potential for error during self administration.^4^ This contrasts with 93% of our patients with CSU who favored this arrangement, and who had real-world experience of transitioning to home administration. Whilst concerns about errors during the injection process remained an issue for our cohort, this was at a comparatively low level. These lower rates of concern in our cohort may be in part due to the training delivered by an experienced nursing team. Our data indicated a high level of patient satisfaction with the training process and a high level of confidence in the self-administration of omalizumab after training was completed.

A frequently cited barrier to home-based omalizumab administration has been the reported risk of anaphylaxis (estimated at 0.2%^5^); however, this was not reported in the key clinical trials investigating the use of omalizumab in CSU.^6^^,^^7^ Individual centres have reported success in transitioning patients with CSU from hospital to home-based omalizumab administration, with similar experiences reflected in a recent multicentre report of 137 patients, where no increase in adverse events or episodes of anaphylaxis was observed.^8^^,^^9^ No studies have specifically focused on the patient experience in this setting, however. Our situation was unique. Given the unanticipated circumstances of the pandemic, we were not afforded months of planning prior to implementing this transition.

Our data indicate that the strong support for home-based treatment amongst this cohort is not solely attributable to the COVID-19 pandemic. Statements regarding drawbacks of receiving omalizumab in hospital drew widespread agreement from respondents, with 86% citing financial costs and 97% citing time commitments as particular disadvantages. Eighty (80%) of patients reported a desire to have commenced self-administration prior to this time. Previous studies and international guidelines on CSU have often cited stress (both physical and emotional) as a trigger for flares of the condition.^2^^,^^10^ It is not implausible that transitioning to home-based therapy (and thus removing burdens of financial cost, long travel times, and the requirement of taking time off work) may itself be a useful intervention in alleviating certain stressors for patients. Outside the context of CSU, benefits associated with self-administered home therapy have been observed in settings as diverse as subcutaneous immunoglobulin for immunodeficiency, depot contraceptive injections and in a variety of rheumatic conditions, with frequently cited advantages of increased patient empowerment, time and cost savings, and reduced psychological burden of disease.^11^, ^12^, ^13^, ^14^

The pandemic forced our team to adapt our restricted use of this effective medication so that patients could continue their treatment with minimal risk.

The COVID-19 pandemic has highlighted a clear rationale for the reduction of unnecessary hospital attendances. Therapies that have previously necessitated hospital-based administration due to funding constraints are certainly not unique to CSU patients. Our data should be viewed encouragingly by other healthcare providers seeking to transition patients to home-based therapies during the pandemic setting. This survey has given an early indication that patients with CSU can manage very successfully in turbulent circumstances with a rapid transition to home-based omalizumab administration and were satisfied with the outcomes achieved. Despite regulatory approval and endorsement in evidence-based guidelines, omalizumab reimbursement, access and administration varies internationally.^15^ Ireland, a wealthy country, has not supported home treatment with omalizumab for patients with CSU. Prescribers internationally may encounter similar restrictions within increasingly constrained resource environments. Our study clearly illustrates to healthcare funders the myriad advantages of facilitating home omalizumab therapy, with cost-saving measures evident for healthcare providers and patients alike. In the expanding landscape of therapeutics for CSU, clinicians ought not be solely focused on the next innovative biologic on the horizon but also look to maximize the benefit that patients can derive from their current treatment. For a sizeable proportion of patients, increased autonomy and improved flexibility by self-administration of omalizumab in the home environment is the logical and timely solution.

## Abbreviations

COVID-19: Coronavirus Disease 2019, CSU: Chronic Spontaneous Urticaria.

## Declaration of competing interest

None.
