# Supplementary material for: Rapid transition to home omalizumab treatment for chronic spontaneous urticaria during the COVID-19 pandemic: A patient perspective
Source: World Allergy Organ J. 2021 Sep 21;14(10):100587. doi: 10.1016/j.waojou.2021.100587 (PMC8452509; doi:10.1016/j.waojou.2021.100587)
Supplement: Multimedia component 1 [file mmc1.docx]

Omalizumab in Chronic Spontaneous Urticaria: Transitioning to Home Administration

Patient Experience Questionnaire

**Consent**

I confirm that (please tick each box if in agreement)

Please tick box

1. I have read the information sheet attached and understand my participation

is voluntary;

(ii) I agree to have my data processed in the method outlined on the information leaflet;

(iii) I agree to partake in the study.

**Patient Questionnaire for Omalizumab (Xolair) home use**

1. Questionnaire respondent
   - I am the patient
   - I am filling out this questionnaire on behalf of the patient
     1. What is your relationship to the patient? __________________

**Please answer the remainder of the questions in relation to the patient, unless otherwise specified.**

1. Gender
   - Male
   - Female
   - Prefer not to answer
2. Age: ______ years
3. For what condition do you receive Omalizumab?
   - Recurrent unprovoked hives (ie. Chronic Spontaneous Urticaria)
   - Recurrent unprovoked swellings (ie. Chronic Spontaneous Angioedema)
   - Recurrent unprovoked hives **and** swellings (Chronic Spontaneous Urticaria-Angioedema)
   - Other: ___________________________________________________
   - Don’t know
4. How long approximately have you been receiving Omalizumab therapy?
   - Less than 1 year
   - 1 – 4 years
   - 5 – 9 years
5. For how long did you have **symptoms** of recurrent hives and/or swelling before starting Omalizumab therapy?
   - Less than 1 year
   - 1 – 4 years
   - 5 – 9 years
   - Greater than 10 years
6. Generally speaking, how satisfied or dissatisfied are you with the way Omalizumab manages your symptoms?
   - Extremely satisfied
   - Satisfied
   - Somewhat satisfied
   - Dissatisfied
   - Extremely dissatisfied
7. In what dosing intervals do you currently receive Omalizumab?
   - 3 weeks
   - 4 weeks
   - 6 weeks
   - Other: ___________________
8. What dose of Omalizumab do you currently receive at each visit?
   - 300mg (2 syringes)
   - Other: _______syringes, or ________mg
9. How flexible would you describe the Omalizumab therapy **in the hospital setting**, prior to the COVID-19 pandemic?
   - Not very flexible
   - Flexible
   - Highly flexible
10. What are your personal expenses for each Omalizumab administration **in the hospital setting**? (public transport costs, private transport – fuel, parking etc)
    - Less than €10
    - €11 - €20
    - €20 - €50
    - Over €50
11. How many days per year do you lose, due to the time taken for your Omalizumab administration? (eg. Taking leave from work for dayward attendances)
    - <1 days
    - 1-10 days
    - 11-20 days
    - >20 days
12. For each dose, approximately how many hours would you save by administering Omalizumab at home?
    - Less than 1 hour
    - 1-5 hours
    - Over 5 hours

**Please indicate the extent to which you agree with the following statements.**

*Please answer the questions by indicating to what extent you agree with the statements. If you have no experience regarding a particular subject, please answer ‘not applicable’.*

General questions

|  | ***Strongly agree*** | ***Partially agree*** | ***Neutral*** | ***Partially disagree*** | ***Strongly disagree*** | *Not applicable* |
| --- | --- | --- | --- | --- | --- | --- |
| **14. I am in favour of injecting Omalizumab by myself at home** |  |  |  |  |  |  |
| **15. For me, the benefits of injecting Omalizumab at home would include:** |  |  |  |  |  |  |
| 1. Cost savings |  |  |  |  |  |  |
| 1. Time savings |  |  |  |  |  |  |
| 1. Greater flexibility in my daily life |  |  |  |  |  |  |
| 1. Less hospital visits |  |  |  |  |  |  |
| 1. Better quality of life |  |  |  |  |  |  |
| 1. Less risk of exposing myself to COVID-19 |  |  |  |  |  |  |
| **16**. **I wish I could have started self-administration sooner** |  |  |  |  |  |  |

Questions relating to practice sessions and contact with the Immunology team

|  | ***Strongly agree*** | ***Partially agree*** | ***Neutral*** | ***Partially disagree*** | ***Strongly disagree*** | *Not applicable* |
| --- | --- | --- | --- | --- | --- | --- |
| **17. The number of practice sessions was sufficient** |  |  |  |  |  |  |
| **18. I felt confident injecting myself after the practice session(s)** |  |  |  |  |  |  |
| **19. The immunology team in the hospital dayward:** |  |  |  |  |  |  |
| 1. Take sufficient time with me |  |  |  |  |  |  |
| 1. Are aware of my situation |  |  |  |  |  |  |
| 1. Answer my questions well |  |  |  |  |  |  |
| 1. Are contactable when needed |  |  |  |  |  |  |

Questions relating to patient concerns

|  | ***Strongly agree*** | ***Partially agree*** | ***Neutral*** | ***Partially disagree*** | ***Strongly disagree*** | *Not applicable* |
| --- | --- | --- | --- | --- | --- | --- |
| **20. I know what to do if I encounter a problem administering the medication** |  |  |  |  |  |  |
| **21. I feel insecure administering the medication** |  |  |  |  |  |  |
| **22. I worry about the following in relation to self-injecting at home:** |  |  |  |  |  |  |
| 1. Injecting myself incorrectly |  |  |  |  |  |  |
| 1. Forgetting to take a dose |  |  |  |  |  |  |
| 1. Developing a side-effect or reaction to the medicine |  |  |  |  |  |  |
| **23. I was concerned about attending the hospital in recent weeks due to the COVID-19 pandemic** |  |  |  |  |  |  |

1. In the future, would you prefer to return to hospital-based treatment? *(Please give a reason for your choice)*
   - Yes
   - No

Please comment below:

1. Since changing to home-based Omalizumab treatment, I am doing:
   1. Much better
   2. Somewhat better
   3. The same
   4. Somewhat worse
   5. Much worse
2. How do you rate the home-based Omalizumab treatment? (0 = very bad, 10 = excellent)
   1. 0 (Very bad)
   2. 1
   3. 2
   4. 3
   5. 4
   6. 5
   7. 6
   8. 7
   9. 8
   10. 9
   11. 10 (Excellent)
3. Would you recommend home-based Omalizumab treatment to other patients with your condition? (0 = definitely not, 10 = definitely yes)
   1. 0 (Definitely not)
   2. 1
   3. 2
   4. 3
   5. 4
   6. 5
   7. 6
   8. 7
   9. 8
   10. 9
   11. 10 (Definitely yes)

**Thank you for completing this questionnaire!**
